# Highly Accessible Atomically Dispersed Fe‐N*_x_* Sites Electrocatalyst for Proton‐Exchange Membrane Fuel Cell

**DOI:** 10.1002/advs.202002249

**Published:** 2021-01-29

**Authors:** Jianing Guo, Bingjie Li, Qiyu Zhang, Qingtao Liu, Zelin Wang, Yufei Zhao, Jianglan Shui, Zhonghua Xiang

**Affiliations:** ^1^ Beijing Advanced Innovation Center for Soft Matter Science and Engineering State Key Laboratory of Organic‐Inorganic Composites Beijing University of Chemical Technology Beijing 100029 P. R. China; ^2^ Hebei Key Laboratory of Inorganic Nanomaterials College of Chemistry and Material Science Hebei Normal University Shijiazhuang Hebei Province 050024 P. R. China; ^3^ Department of Oncology The First Affiliated Hospital Zhengzhou University 1 Jianshe Street Zhengzhou Henan 450052 P. R. China; ^4^ School of Materials Science and Engineering Beihang University Beijing China; ^5^ State Key Laboratory of Chemical Resource Engineering Beijing University of Chemical Technology Beijing 100029 P. R. China

**Keywords:** acidic media, covalent organic polymer, oxygen reduction reaction, proton exchange membrane fuel cells, single‐atom catalysts

## Abstract

Atomically dispersed transition metal‐N*_x_* sites have emerged as a frontier for electrocatalysis because of the maximized atom utilization. However, there is still the problem that the reactant is difficult to reach active sites inside the catalytic layer in the practical proton exchange membrane fuel cell (PEMFC) testing, resulting in the ineffective utilization of the deeply hided active sites. In the device manner, the favorite structure of electrocatalysts for good mass transfer is vital for PEMFC. Herein, a facile one‐step approach to synthesize atomically dispersed Fe‐N*_x_* species on hierarchically porous carbon nanostructures as a high‐efficient and stable atomically dispersed catalyst for oxygen reduction in acidic media is reported, which is achieved by a predesigned hierarchical covalent organic polymer (COP) with iron anchored. COP materials with well‐defined building blocks can stabilize the dopants and provide efficient mass transport. The appropriate hierarchical pore structure is proved to facilitate the mass transport of reactants to the active sites, ensuring the utilization of active sites in devices. Particularly, the structurally optimized HSAC/Fe‐3 displays a maximum power density of up to 824 mW cm^−2^, higher than other samples with fewer mesopores. Accordingly, this work will offer inspirations for designing efficient atomically dispersed electrocatalyst in PEMFC device.

## Graphical abstract

A facile one‐step approach to synthesize atomically dispersed Fe‐N*_x_* species on hierarchically porous carbon nanostructures is reported, which facilitates the mass transport of reactants and electrolytes to the active sites, thus ensuring the efficient utilization of active sites in practical PEMFC devices.

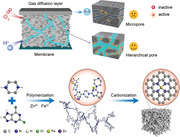

The insufficient local mass transport and poor stability of nonprecious metal catalysts (NPMCs) restrict the commercial applications of proton exchange membrane fuel cell (PEMFC).^[^ ^1^ ^]^ The current test method of measuring oxygen reduction reaction (ORR) performance is to use the rotating disk electrode (RDE) which provides an idealized experimental condition with negligible influence of mass transport.^[^ ^2^ ^]^ However, the membrane electrode assembly (MEA) performance is limited by the inability of reactants to reach the active sites inside the catalytic layer in the practical testing of MEA.^[^ ^3^ ^]^ Meanwhile, many efficient NPMCs are prepared using micropores dominated precursors to maximize the density of active sites and thus increase ORR activity.^[^ ^4^ ^]^ The micropores are important to host active sites for ORR, but tremendous active sites are hided insides the catalyst bulk, which further exacerbates the insufficient local mass transport of both reactant (O_2_) and product (H_2_O) to and from the active sites.^[^ ^5^ ^]^ Moreover, the accumulation of water in practical PEMFC would also cause ineffective utilization of the hided active sites (**Figure** **1**a).^[^ ^6^ ^]^ Hence, it is essential to improve mass transport to increase the accessible area of reactant for catalytic applications of NPMCs.

**Figure 1:**
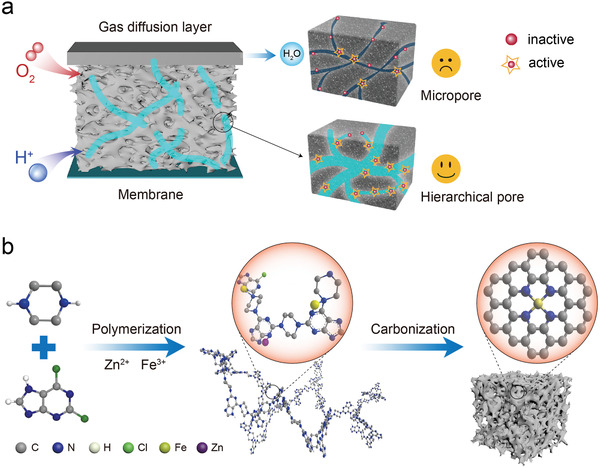
a) Schematic illustration of pore structure of catalytic layer. b) A schematic synthesis process of catalyst.

It is generally acknowledged that hierarchically porous structures can form ideal channels for reactants and electrolytes into the active sites.^[^ ^7^ ^]^ Especially, mesoporous and macroporous structures with suitable pore architectures can facilitate efficient mass transport inside catalysts, improving the utilization of active sites (Figure 1a).^[^ ^8^ ^]^ Therefore, the porous structure of carbon support should be rationally designed, which can effectively improve catalyst performance.^[^ ^9^ ^]^ Recently, due to its high thermal stability and precisely controllable capacities, covalent organic polymer (COP) attract tremendous interest as a class of ever‐growing porous materials.^[^ ^10^ ^]^ COP materials exhibit robust tailoring heteroatom incorporation, plentiful pore structure, and versatility similar to metal‐organic framework (MOF).^[^ ^11^ ^]^ Compared with the extensively studied micropore dominated zeolitic imidazolate framework‐8 (ZIF‐8),^[^ ^12^ ^]^ one of stable MOF materials, COP materials could design adjustable hierarchically porous structures through tailoring length and geometry of organic linkers in the molecular manner, which may provide rapid proton adsorption, reduction, and a product release.^[^ ^13^ ^]^

Here, we presented a one‐step synthesis of hierarchically porous carbon with atomically dispersed Fe‐N*_x_* species as high‐efficient, low‐cost, and stable electrocatalysts for ORR in acidic media, which was derived from a meso/macro‐pores dominated COP material via a hydrothermal method and followed by calcination. The rich nitrogen coordination sites in COP materials could stabilize single‐atom metals to enhance the activity and stability of catalysts.^[^ ^14^ ^]^ Meanwhile, the zinc (Zn) evaporation during the pyrolysis not only facilitates the pore‐forming of micropores and mesopores, but also can isolate iron (Fe) to suppress its agglomeration during the sintering process.^[^ ^12^ ^]^ The appropriate hierarchically porous structure enhances the exposure of active sites and facilitates mass transport, thus ensuring the efficient utilization of active sites. Through adjusting the Zn contents doped in the polymer, we investigated the effect of porous structure on the ORR performance. The structurally optimized catalyst exhibits great electrocatalytic activities and stabilities for ORR in acidic media. In a practical PEMFC device, the catalyst gave an outstanding performance with a high maximum power density of 824 mW cm^−2^, significantly higher than samples with fewer mesopores. Therefore, mesoporous COP materials for the first time facilitate the preparation of highly efficient single atom catalysts (SACs) for PEMFC application.

Typically, hierarchical single‐atom catalysts/Fe‐*X* (termed as HSAC/Fe‐*X*, *X* is moles of zinc added in the original solution) are synthesized via a facile hydrothermal process with 2,6‐Dichloropurine, piperazine, Zn(NO_3_)_2_·6H_2_O and FeCl_3_·6H_2_O, followed by calcination (Figure 1b). The Fourier transform infrared spectroscopy and Solid‐state ^13^C NMR indicate the successful synthesis of COP‐PD (Figures S1 andS2, Supporting Information). From the powder X‐ray diffraction patterns analysis in **Figure** **2**a and Figure S3, Supporting Information, we can clearly observe the material structural change after the calcination. The broad peak centered at 23.4° of origin COP may be assigned to the amorphous reflection (001) of aromatic sheets, while other diffraction peaks at 31.7°, 34.4°, 36.3°, 47.5°, 56.6°, 62.9°, and 67.9° can be indexed to the lattice plane of (100), (002), (101), (102), (110), (103), and (112) of the ZnO (JCPDS No.05‐0664), respectively (Figure S3, Supporting Information). The ZnO can be reduced to metallic Zn at the temperatures above 800 °C. Metallic Zn will evaporate at the temperature above 907 °C,^[^ ^15^ ^]^ which serves as a pore‐forming agent for micropores and mesopores during the carbonization process and also suppresses Fe agglomeration.^[^ ^16^ ^]^ After high‐temperature pyrolysis, there are only two broad peaks at about 26.1° and 42.9° for three catalysts (Figure 2a), corresponding to the (002) and (101) crystal planes of graphitic carbon, respectively.^[^ ^17^ ^]^ X‐ray photoelectron spectroscopy (XPS) was used to analyze the chemical composition and valance state of the catalysts. The high‐resolution N 1s spectra of HSAC/Fe‐2, HSAC/Fe‐3, and HSAC/Fe‐4 catalysts were displayed in Figure 2b. The spectra could be fitted well with four primary peaks, which assigned to the pyridinic N (398.2 eV), Fe‐N*_x_* (398.9 eV), pyrrolic N (400.4 eV), graphitic N (401.2 eV), respectively, indicating the existence of Fe‐N*_x_* coordination structure in the catalyst.^[^ ^18^ ^]^ The relative contents of different types of N are shown in Figure S4a, Supporting Information. Pyridinic N can serve as anchor sites for iron atoms and decrease the ORR overpotential, while the graphitic N can improve the electron transfer of the catalyst especially in the limit current density region.^[^ ^19^ ^]^ Figure S4b, Supporting Information shows the high‐resolution scan of Fe 2p for three catalysts, where the deconvolution yields two pairs of peaks for Fe^2+^ (710.9 and 724.1 eV) and Fe^3+^ (716.2 and 731.6 eV).^[^ ^20^ ^]^ The surface Fe contents of three catalysts (HSAC/Fe‐2, HSAC/Fe‐3 and HSAC/Fe‐4) are 1.39 1.29 and 1.75 wt%, respectively (Table S1, Supporting Information), while the inductively coupled plasma optical emission spectrometry (ICP‐OES) characterization indicates that the three catalysts themselves have higher Fe contents (3.29, 2.78, and 3.37 wt%, respectively) (Table S2, Supporting Information), which indicates that there are many single‐atom Fe distributed inside the catalysts. That is to say that it is important to efficiently utilize these hidden single Fe sites, for example, the above mentioned dispersed on an appropriate accessble hierarchically porous structure (Figure 1a), in practical PEMFCs.

**Figure 2:**
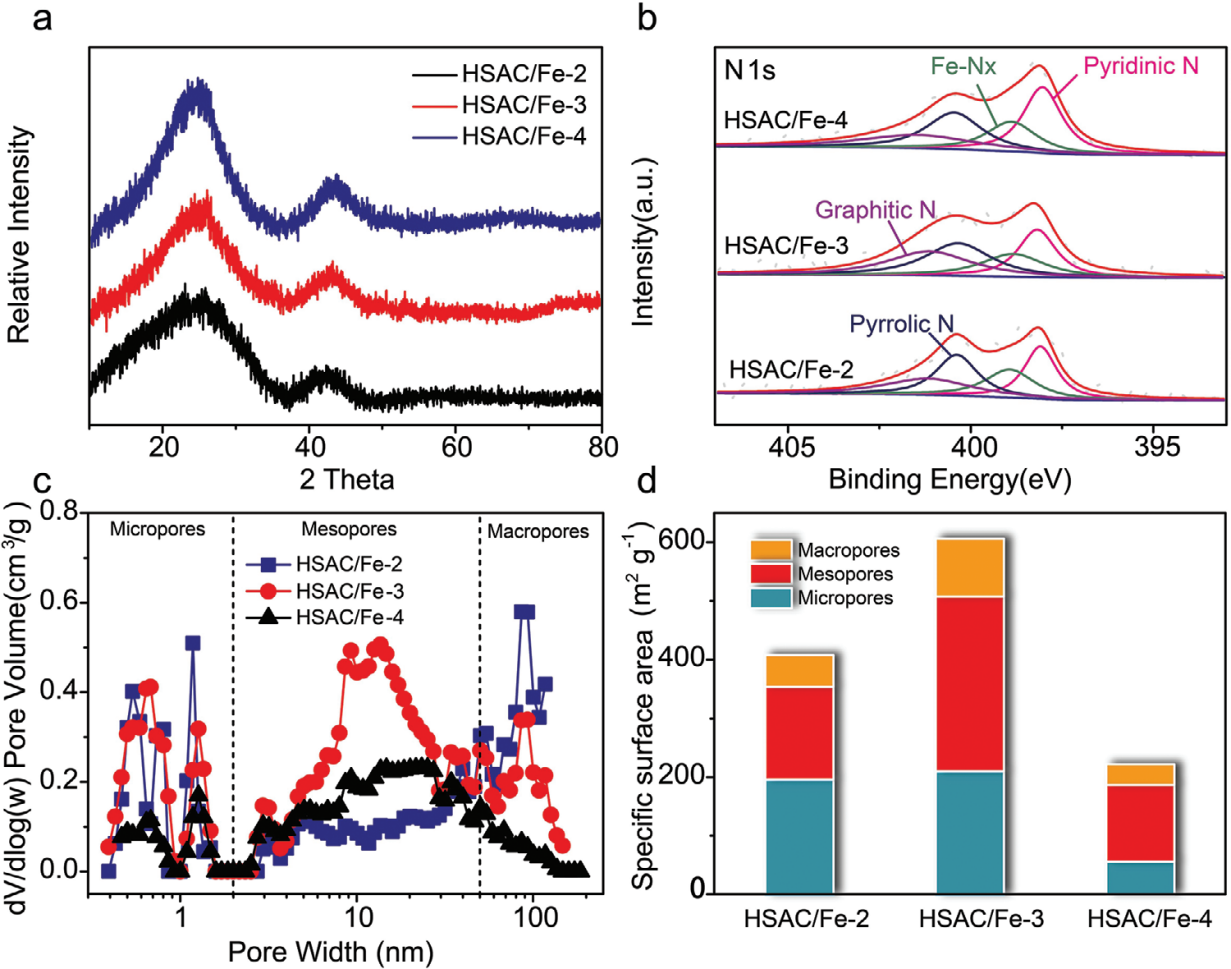
Composition and structure characterization of HSAC/Fe‐2, HSAC/Fe‐3, and HSAC/Fe‐4 catalysts. a) XRD patterns. b) High‐resolution XPS spectra of N1s. c) The pore size distributions of the three HSAC/Fe samples. d) The comparison of surface area of micropore, mesopore, and macropore for the catalysts.

Nitrogen adsorption‐desorption isotherms were measured to gain deep insight into the structure feature of catalysts. As can be seen from Figure S5, Supporting Information, the COP‐Fe‐Zn itself has a certain mesoporous and macroporous structure, conducing to the formation of hierarchical pores. After carbonization, the Brunauer–Emmett–Teller (BET) specific surface area of HSAC/Fe‐3 catalyst is calculated to be 606.7 m^2^ g^−1^, higher than that of HSAC/Fe‐2 (407.7 m^2^ g^−1^) and HSAC/Fe‐4 (221.8 m^2^ g^−1^) (Table S3 and Figure S6, Supporting Information). All catalysts exhibit a typical type IV isotherm curve with a hysteresis loop at intermediate pressure, demonstrating the presence of mesoporous structure.^[^ ^21^ ^]^ The pore size distribution in Figure 2c clearly shows the hierarchical feature of the resultant catalysts. The micropores were generated by the evaporation of Zn during the pyrolysis, while the meso/macro‐pores are associated with the mesopore and macroporous of the pristine COP‐Fe‐Zn. This is the key difference between COP and commonly used ZIF‐8 templates that usually produce micropore dominated Fe‐N‐C SACs.^[^ ^9c^ ^]^ Especially, HSAC/Fe‐3 catalyst displays more drastic increase on the curve at low pressure (*P*/*P*_0_ < 0.01), indicating the rich of micropores which may expose more active sites and stabilize single metal atoms.^[^ ^9^, ^22^ ^]^ Meanwhile, the HSAC/Fe‐3 catalyst possesses rich mesopores, much higher than HSAC/Fe‐2 and HSAC/Fe‐4. Figure 2d summarized the specific surface areas of each catalyst. These mesopores and macropores are conducive to the accessible permeation of oxygen and efflux of products, which will facilitate the mass transfer and improve the kinetics of the ORR catalysis process.^[^ ^23^ ^]^ The following electrochemical test results will indicate that increasing the proportion of mesoporous is more advantageous than increasing the proportion of micropore.

The morphology and microstructure of catalysts were characterized by scanning electron microscopy, high‐resolution transmission electron microscopy (HRTEM) and scanning transmission electron microscopy (STEM). As is shown in Figure S7, Supporting Information, the HSAC/Fe‐2, HSAC/Fe‐3, and HSAC/Fe‐4 catalysts have a porous structure without noticeable metal aggregation, while there are obvious Fe agglomerated particles on catalyst HSAC/Fe‐0, which indicates that Zn evaporation could effectively prevent the agglomeration of Fe metal particles. The HRTEM image shows a mesoporous structure of the catalyst that should be derived from the porous COP precursor. From the annular bright field STEM image (Figure S8, Supporting Information), it can be seen that there are clear graphite‐like carbon lattice bands in flake‐like structures of HSAC/Fe‐3 catalyst, which indicates a good conductivity of the catalyst conducive to the charge transfer in the electrocatalytic process. Furthermore, the aberration‐corrected dark‐field (HAADF)‐STEM was further conducted to investigate the atomic Fe sites in the HSAC/Fe‐3 catalyst. A number of bright dots with homogeneously atomic dispersion in the porous carbon substrate for HSAC/Fe‐3 catalyst unambiguously appear, which could be ascribed to the heavier Fe single atoms (**Figure** **3**a). Iron and nitrogen atoms were further identified and mapped out by electron energy loss spectroscopy (EELS) analysis (Figure 3b). Specifically, the signals of carbon, nitrogen, and iron are distinguishable in EELS spectrum. The corresponding EELS mappings reveal the densely and atomically dispersed Fe single‐atoms on the carbon substrate.

**Figure 3:**
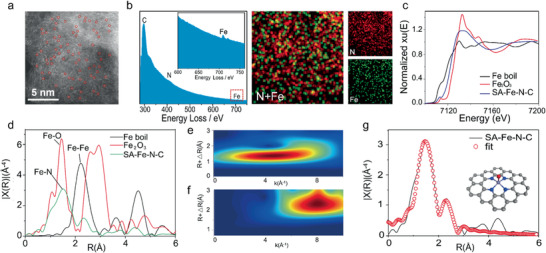
a) High‐resolution HAADF‐STEM image, b) EELS spectrum of HSAC/Fe‐3 and EELS mapping of nitrogen, iron, and overlaid iron and nitrogen for the HSAC/Fe‐3, c) Fe K‐edge XANES, d) Experimental Fourier Transform at the Fe K‐edge of EXAFS data of HSAC/Fe‐3, Fe foil, and Fe_2_O_3_ as references. The wavelet transform (WT) of e) HSAC/Fe‐3 catalyst and f) Fe foil. g) The corresponding EXAFS r space fitting curves of HSAC/Fe‐3 (insert image is the schematic model of HSAC/Fe‐3, Fe (purple), N (blue), O (red), and C (gray)).

X‐ray absorption near‐edge structure (XANES) and X‐ray absorption fine structure (EXAFS) were probed to further prove the atomic dispersion of Fe species and structural information of the coordination environment of HSAC/Fe‐3 catalyst. As shown in the XANES of HSAC/Fe‐3 with Fe foil and Fe_2_O_3_ as references (Figure 3c), the absorption edge of HSAC/Fe‐3 is between that of Fe foil and Fe_2_O_3_, indicating the Fe atom valence is situated between Fe^0^ and Fe^3+^.^[^ ^24^ ^]^ The EXAFS spectrum of HSAC/Fe‐3 in Figure 3d just shows one main peak at 1.51 Å, corresponding to the Fe‐N/C first coordination shell. No obvious Fe—Fe peak (2.2 Å) was detected that is in agreement with the HAADF‐STEM result, indicating all the Fe species are atomically dispersed without aggregation.^[^ ^25^ ^]^ Wavelet transform (WT) of Fe K‐edge EXAFS oscillations was carried out to more clearly indicate the atomic dispersion of iron of HSAC/Fe‐3. Impressively, there is only one WT intensity maximum at about 5 Å^−1^ for HSAC/Fe‐3 (Figure 3e), which could be assigned to the Fe—N(O) bonding. No intensity maximum associated with Fe—Fe is detected compared with the WT plots of Fe foil (Figure 3f).^[^ ^26^ ^]^ Therefore, FT‐ and WT‐EXAFS analysis demonstrates that iron atoms were atomically dispersed in HSAC/Fe‐3. According to EXAFS fitting results in Figure 3g and Table S4, Supporting Information, the coordination number of Fe was about 5 and the mean bond length was 2.01 Å. Based on the above analysis, the local structure around Fe was constructed. The isolated Fe atom was atomically anchored in the nitrogen doped porous carbon matrix and was fourfold coordinated by N atoms. It should be mentioned that one O_2_ molecule might be adsorbed on the Fe atom in perpendicular to Fe‐N_4_ plane (Figure 3g).^[^ ^26^ ^]^

The ORR performances of the prepared catalysts were investigated by the rotating disk electrode (RDE) in O_2_‐saturated 0.5 m H_2_SO_4_ solution at room temperature, and the commercial Pt/C catalyst was used for comparison. As is shown in **Figure** **4**a, compared with HSAC/Fe‐2 and HSAC/Fe‐4 catalysts, HSAC/Fe‐3 catalyst exhibits the highest ORR catalytic activity with an onset potential (*E*_onset_) of 0.94 V and half‐wave potential (*E*_1/2_) of 0.814 V, much higher than HSAC/Fe‐2 (*E*_onset_ = 0.90V, *E*_1/2_ = 0.775V) and HSAC/Fe‐4 (*E*_onset_ = 0.87V, *E*_1/2_ = 0.692V). The *E*_1/2_ of HSAC/Fe‐3 catalyst is only 13 mV lower than that of commercial Pt/C catalyst and in line with that observed in current state‐of‐the‐art non‐precious ORR catalysts (Figure S9a, Supporting Information), manifesting its promising potential as alternatives to noble‐metal catalysts in acidic media (Table S5, Supporting Information). Meanwhile, the mass activity of HSAC/Fe‐3 catalyst achieves 52.7 mA mg^−1^ at 0.75 V, about 3.5 times and 14 times higher than that of HSAC/Fe‐2 (14.6 mA mg^−1^) and HSAC/Fe‐4 (3.5 mA mg^−1^), respectively, almost close to that of Pt/C (55.7 mA mg^−1^) (Figure S9b, Supporting Information), which is consistent with the results of the pore size distribution for catalysts. The rotating ring disk electrode measurements demonstrate that the best performing HSAC/Fe‐3 catalyst generates the lowest hydrogen peroxide (H_2_O_2_) yield below 2.5% in the potential range of 0.2 to 0.85 V, even lower than that of Pt/C (Figure S10, Supporting Information). The corresponding electron transfer number of HSAC/Fe‐3 also reveals a close‐to‐four electron reduction pathway, indicating a high selectivity of 4e^−^ oxygen reduction reaction (Figure S10, Supporting Information). Moreover, HSAC/Fe‐3 catalyst shows a Tafel slope of 67 mV dec^−1^ that is same as that of Pt/C and much lower than those of HSAC/Fe‐2 (77 mV dec^−1^) and HSAC/Fe‐4 (97 mV dec^−1^), suggesting the superior catalytic kinetic process of HSAC/Fe‐3 for ORR (Figure 4b).

**Figure 4:**
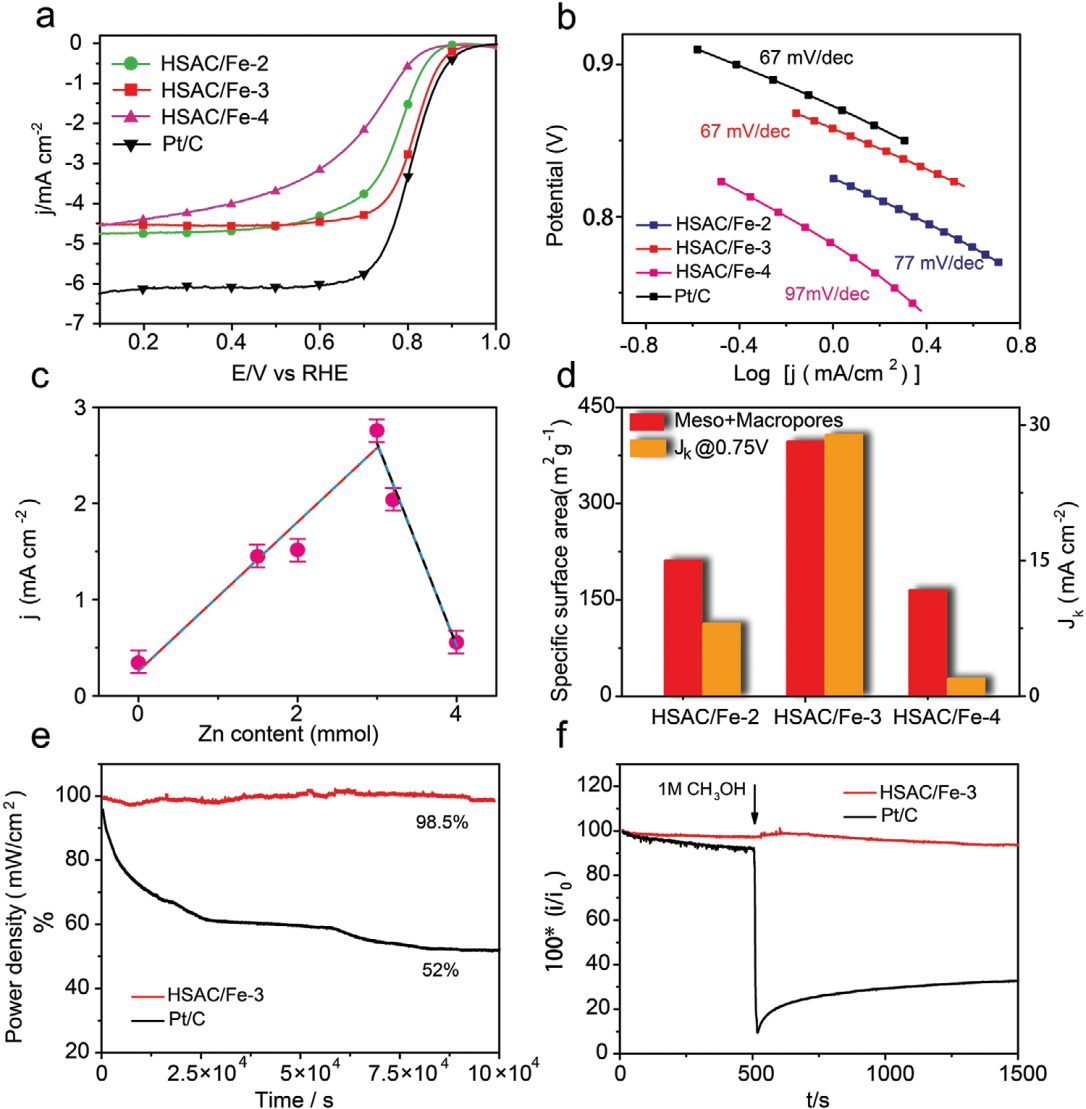
a) ORR polarization curves, b) the corresponding Tafel plots of HSAC/Fe‐2, HSAC/Fe‐3 and HSAC/Fe‐4 in O_2_‐saturated 0.5 m H_2_SO_4_ solution at 1600 rpm, Pt/C in 0.1 m HClO_4_ at 1600 rpm as a comparison, c) the “volcano plot” correlation between Zn incorporation in the COP precursors and activity of final pyrolytic catalysts reflected by the current density at 0.8 V, d) the sum specific surface area of mesopores and macropores, *J*_k_ at 0.75 V, e) long‐term stability of HSAC/Fe‐3 and Pt/C by Chronoamperometric, f) methanol crossover effect test of HSAC/Fe‐3 catalyst and Pt/C (20%) upon addition of methanol (1 m) at 0.55 V (versus RHE) after around 500 s.

In order to further study the effects of Zn evaporation as to the final pyrolytic catalysts, ORR polarization plots of the catalysts with different Zn incorporation in the COP precursors were recorded (Figure S11, Supporting Information). Interestingly, the effect of Zn content on ORR performance presents a so‐called “volcano plot” (Figure 4c). It could be easily noted that the catalyst (HSAC/Fe) derived from COP without Zn exhibits a poor activity. Zn incorporation in the COP precursor plays a significant role in facilitating ORR activity of the final catalyst, which is likely associated with the formation of micropores and mesopores on catalyst due to Zn evaporation during the pyrolysis process. It is worth noting that too much Zn doping may lead to the destruction of carbon structures with insufficient micropore and small specific surface area, which results in inadequate active sites and a decrease in ORR activity of catalyst. Obviously, HSAC/Fe‐3 has the optimized Zn doping content of 3 mmol and optimal porosity, thus exhibits the best ORR activity among the three samples. As shown in Figure 4d and Figure S12, Supporting Information, HSAC/Fe‐3 catalyst shows a high kinetic current density (*J*_k_) of 28.96 mA cm^−2^ at 0.75 V, much surpassing that of the HSAC/Fe‐2 (8.044 mA cm^−2^) and HSAC/Fe‐4 catalyst (1.95 mA cm^−2^). This sequence consists with the surface areas of mesopores and macropores of catalysts, indicating that the porosity of the catalyst is a crucial factor for ORR activity of non‐precious metal catalysts, and the hierarchically porous structure is favorable to promote ORR. Furthermore, catalyst stability was studied using chronoamperometry test at a constant potential of 0.35 V in O_2_‐saturated 0.5 m H_2_SO_4_. The HSAC/Fe‐3 catalyst exhibits a greatly encouraging stability with a 98.5% current retention after 100 000 s (Figure 4e), significantly higher than the 52.0% retention of Pt/C. The long‐term durability can be ascribed to the fact that the single‐atom Fe species were stabilized in the unique porous structure of HSAC/Fe‐3 catalyst. Furthermore, there is no obvious change in the current density of HSAC/Fe‐3 catalyst after introducing 1.0 m methanol into the electrolyte (Figure 4f), indicating that HSAC/Fe‐3 catalyst is nearly free from the methanol crossover effect, nevertheless the current density for Pt/C has a dramatic decrease.

Fuel cell tests were further conducted to evaluate the performance of the three HSAC/Fe catalysts. 5 cm^2^ membrane assembly electrode (MEA) with a total catalyst loading of 3.0 mg cm^−2^ was tested under absolute H_2_/O_2_ pressures of 2 bar in a single cell fixture. HSAC/Fe‐3 catalyst achieves a high maximum power density (*P*_max_) of 824 mW cm^−2^ at 0.46 V, much higher than 448 mW cm^−2^ of HSAC‐2 and 498 mW cm^−2^ of HSAC/Fe‐4 (**Figure** **5** and Figure S13, Supporting Information). Compared with other catalysts for MEA performance, HSAC/Fe‐3 catalyst still shows high MEA performance and good stability(Table S6 and Figure S14, Supporting Information). It is noteworthy that HSAC/Fe‐4 exhibited better performance than HSAC/Fe‐2, especially in the ohmic polarization and concentration polarization stages, which were contrary to their ORR activity (Figures 4a and 5; Table S3, Supporting Information). This reflects the importance of mesopore in the catalysts to promote mass transfer in PEMFC. Referring to the porosity characteristics of three catalysts, these cell performances clearly demonstrate that the hierarchically porous structure with appropriate micropores and mesopores can significantly improve the fuel cell power performance of non‐precious metal catalysts.

**Figure 5:**
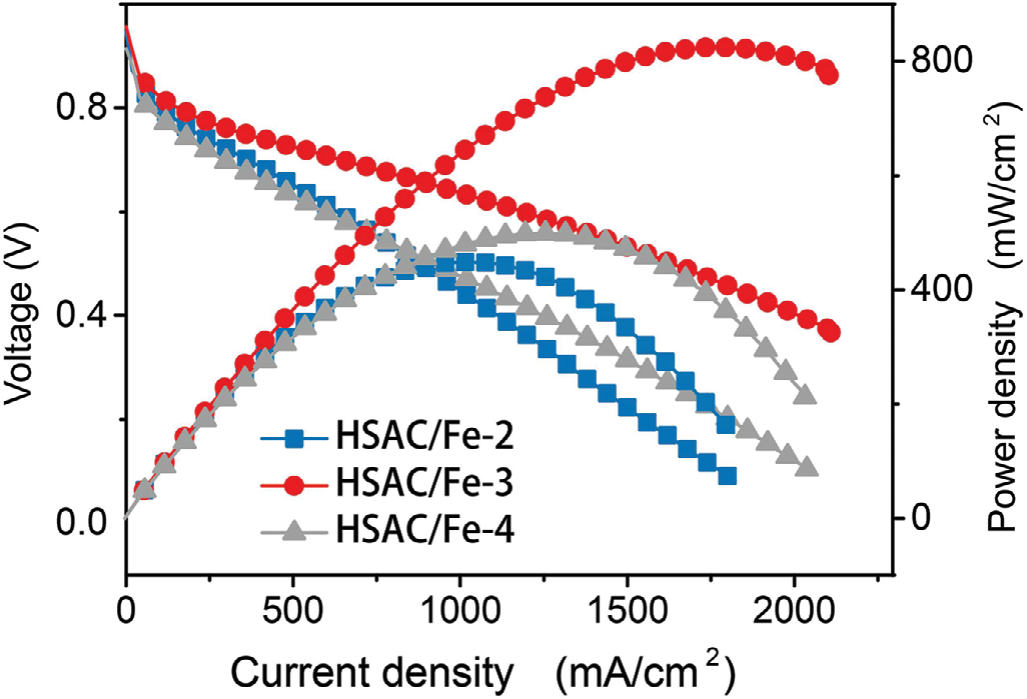
*I*–*V* polarization and power density curves of HSAC/Fe‐2, HSAC/Fe‐3 and HSAC/Fe‐4 with cathode loadings of 3 mg cm^−2^ and anode loading of 0.33 (Pt) mg cm^−2^, respectively. PEMFC tests were applied in a 5 cm^2^ single cell with 80 °C H_2_/O_2_ at flow of 0.3 L min^−1^ for H_2_ and 0.4 L min^−1^ for O_2_, 100% relative humidity (RH), 2 bar absolute pressure.

In summary, based on a meso/macro‐pore dominated COP material, we have developed a facile one‐step method to prepare the hierarchically porous carbon with atomically dispersed Fe‐N*_x_* species as high‐efficient and stable electrocatalysts for ORR in acidic media. The pyridinic nitrogen sites from the pristine COP structure could act as anchoring points to stabilize Fe single atoms, preventing metal agglomeration and enhancing the catalyst stability. Our results indicate that a high proportion of mesopores show more advantageous than that of micropore for the PEMFC power performance. The appropriate hierarchically porous structure is proved efficient to facilitate the mass transport of reactants and electrolytes to the active sites, thus ensuring the efficient utilization of active sites in devices. Particularly, the structurally optimized HSAC/Fe‐3 catalyst shows excellent catalytic activity for ORR with a half‐wave potential of 0.814 V as well as remarkable long‐term durability with a 98.5% current retention after 100 000 s in acid media. In PEMFCs, HSAC/Fe‐3 displays a high power density up to 824 mW cm^−2^, much better than other control samples with less mesopores. Accordingly, this work will offer positive inspirations for designing and fabricating highly efficient non‐precious metal electrocatalysts for various renewable energy applications besides fuel cells.

## Conflict of Interest

The authors declare no conflict of interest.

## Supporting information

Supporting Information
