# Supplementary material for: Highly Accessible Atomically Dispersed Fe‐Nx Sites Electrocatalyst for Proton‐Exchange Membrane Fuel Cell
Source: Adv Sci (Weinh). 2021 Jan 29;8(5):2002249. doi: 10.1002/advs.202002249 (PMC7927611; doi:10.1002/advs.202002249)
Supplement: Supplementary file 1 — Supporting Information [file ADVS-8-2002249-s001.pdf]

WILEY-VCH

© 2021 Wiley-VCH GmbH

ADVANCED SCIENCE
Open Access

Supporting Information

for *Adv. Sci.*, DOI: 10.1002/advs.202002249

Highly accessible atomically dispersed Fe-N_x sites electrocatalyst for proton-exchange membrane fuel cell

Jianing Guo, Bingjie Li, Qiyu Zhang, Qingtao Liu, Zelin Wang, Yufei Zhao, Jianglan Shui,* and Zhonghua Xiang**

Supporting Information

Materials

All reagents were obtained from commercial sources. 2,6-Dichloropurine and N,N-diisopropylethylamine (DIEA) were purchased from Bide Pharmatech Ltd. and J&K, respectively. Tetrahydrofuran (THF) and zinc(II) nitrate hexahydrate ($Zn(NO_3)_2 \cdot 6H_2O$) were obtained from Beijing Chemical Works. Piperazinewere and Ferric chloride hexahydrate ($FeCl_3 \cdot 6H_2O$) were purchased from Aladdin. 5% Nafion were obtained from Asta Tech and DuPont. Commercial Pt-based catalyst was supplied by Alfa Aesar Chemical Co.Ltd. High-purity argon and oxygen gas was provided from Beijing AP BAIF Gases Industry Co. Ltd.

Synthesis

During the synthesis, a wide range of Fe-doped COP was synthesized by tuning the concentration of Zn content in solution. For convenience, the precursors are designated as COP-PD-n, where n is defined as the molar number of Zn content in the starting solution. Here the n value varied from 0 (Zn-free) to 4 mmol. Typically, 1.5 mmol 2,6-Dichloropurine, 3 mmol $Zn(NO_3)_2 \cdot 6H_2O$ and 200 ul $FeCl_3$ $6H_2O$ (50 mg/mL) were dissolved in 70 mL THF with ultrasonic. Then the above solution was stirred in an ice bath. When the temperature of the solution dropped to 0 °C, 3 mmol piperazine and 4.25 ml DIEA were added and continued to stir for 4 h at 0 °C. After the temperature of this solution naturally returned to room temperature, the solution was heated to 90 °C and reacted with stirring at this temperature for 12 h. The obtained product was separated by filtration and washed with water. Finally, the product was dried at 60 °C under vacuum for overnight, which was called as COP-PD-3.

The power of COP-PD-n was then transferred into a ceramic boat and placed in a tube furnace. The sample was was heated to 975 °C and kept at 975 °C for 2 h under Ar gas. Finally, the sample was naturally cooled to room temperature. The resultant was named as HSAC/Fe-n, where n is molar number of Zn content in the starting solution.

Physical Characterization

The scanning electron microscopy (SEM) images were taken by using a S-4700 instrument. The high-resolution transmission electron microscopy (HRTEM) images were obtained on a 2100F instrument. The high-angle annular dark-field scanning transmission electron microscopy (HAADFSTEM) images were taken by using a JEM-ARM 300F

scanning/transmission electron microscope operated at 300 kV, equipped with a probe spherical aberration corrector. Powder X-ray diffraction (PXRD) measurements were characterized by D/MAX 2000 X-ray diffractometer with Cu Kα line (λ=1.54178 Å) as the incident beam. X-ray photoelectron spectroscopy (XPS) analysis was investigated on ThermoVG ESCALAB 250 using Al Kα irradiation. Nitrogen adsorption-desorption isotherms were measured by ASAP 2460. FT-IR spectroscopy was performed on an VERTEX 70V instrument with the wavenumber range of 400-4000 cm^{-1}. Fe K-edge X-ray absorption spectra were performed on the on the 1W1B-X-ray absorption spectrum experiment stations of Beijing Synchrotron Radiation Facility, China, operated at the energy of 2.5 GeV with an average electron current of 250 mA. A Si (111) double-crystal monochromator was used to reduce the harmonic component of the monochrome beam. The Fe K-edge XANES data were recorded in a transmission mode. Fe foil and Fe3O4 were used as references. The acquired EXAFS raw data were background-subtracted, normalized and Fourier transformed according to the standard procedures with the IFEFFIT software package. The k3 weighted extended EXAFS oscillation in the range of 3-10.8 Å-1 was Fourier-transformed (FT). The Solid-state ^{13}C NMR was performed on a AV300 instrument. The inductively coupled plasma emission spectroscopy (ICP-OES) was performed on ICPS-7500.

Electrochemical measurements

All the electrochemical measurements were carried out in a conventional three-electrode cell using CHI 760E electrochemical workstation controlled equipped with a glassy carbon rotating disk electrode (RDE, 5 mm diameter, PINE instrument Inc.) at room temperature. The electrochemical measurements were conducted in 0.5 M H_2SO_4 electrolyte using the catalyst modified glassy carbon electrode as the working electrode, saturated calomel electrode (SCE) as the reference electrode and 0.1 cm^2 platinum foil as the counter electrode. All potentials reported in this work were calibrated to the reversible hydrogen electrode (RHE). The typical ink included 5 mg electrocatalyst ,100 μL of Nafion solution (0.5 wt.%) and 1 mL of anhydrous ethanol. Subsequently, they were ultrasonic for about 30 minutes to form a homogeneous solution. Then, 25 μL of the as-prepared catalyst ink was dropped on a glassy carbon rotating disk electrode giving a loading of 0.57 mg cm^{-2}. The commercially carbon-supported Pt/C electrode was prepared according to the above procedure with same loading. Electrolyte (0.5 M H_2SO_4) was saturated with high-purity oxygen at room temperature by bubbling O_2 prior to the measurements for at least 30 mins. A flow of O_2 was maintained over the electrolyte during

the test. The working electrode was cycled at least 25 times before the datas were recorded at a scan rate 100 mV·s^{-1}.The linear sweep voltammetry (LSV) for ORR was performed at a scan rate of 5 mV s^{-1} under 1600 rpm.

The electron transfer number and peroxide percentage were tested by the rotating ring disk electrode (RRDE) measurements. The electron transfer number (n) was determined by the followed equation:

$$n = 4 \times \frac{I_d}{I_d + I_r / N} \qquad \textbf{(SQ1)}$$

The ring potential was constant at 1.25 V vs. RHE to reduce H_2O_2. The peroxide percentage (% HO_2^-) was calculated based on the equation:

$$\%HO_2^- = 200 \times \frac{I_r / N}{I_d + I_r / N} \qquad \textbf{(SQ2)}$$

where I_d is disk current, I_r is ring current and N = 0.37 is the current collection efficiency of the Pt ring.

The kinetic current density (J_K) was calculated from the Koutecky-Levich equation:

$$\frac{1}{J} = \frac{1}{J_L} + \frac{1}{J_K}$$

where J is the measured current density, J_K and J_L are the kinetic and limiting current densities, respectively.

MEA Preparation and PEM Fuel Cell Tests

The catalyst was mixed with Nafion solution (5 wt. %), isopropanol and deionized water to prepare the catalyst ink, which contained the same weight of Nafion ionomer as the catalyst. The ink was then subjected to a sonication for 20 min. The well-dispersed ink was brushed on a piece of carbon paper (5 cm^2) as cathode with loading of ~3 mg cm^{-2}, followed by a drying in vacuum at 80 °C for 2 h. As for anode, Pt/C (20 wt. % of Pt, BASF) was used with loading of ~0.35 mg cm^{-2}. The prepared cathode and anode were pressed onto the two sides of a Nafion 211 membrane (DuPont) at 130 °C for 90 seconds under a pressure of 1.5 MPa to obtain the MEA. The MEA was measured by a fuel cell test station (Scribner 850e) with UHP-grade H_2 and O_2 at 80 °C. All the gases were humidified at 80 °C with the flow rate of 0.3 L min^{-1} for H_2 and 0.4 L min^{-1} for O_2. The absolute pressures of H_2 and O_2 were the same and set at 2 bar. The flow rate for the durability test was reduced to 0.1 L min^{-1}for both gases.

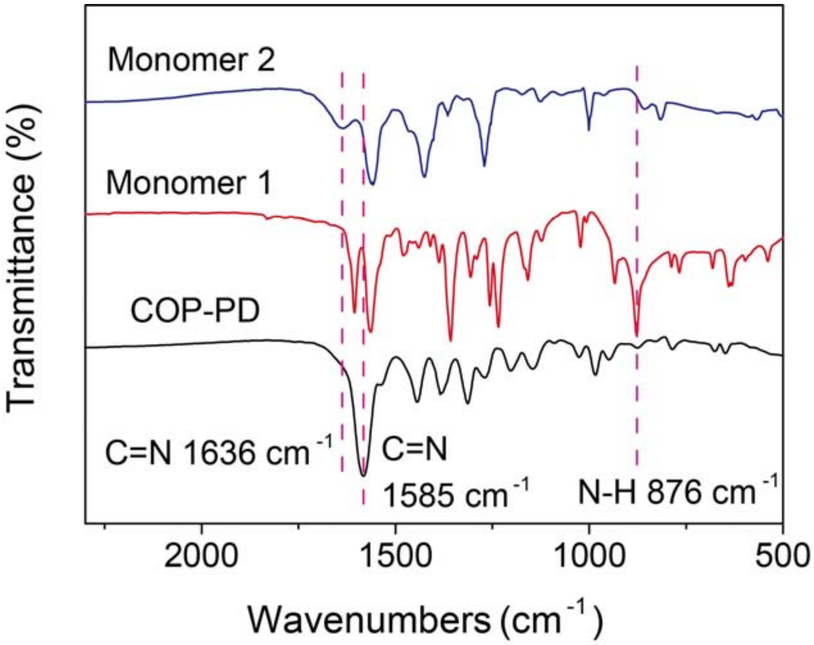

Figure S1. The FT-IR spectra of COP-PD. Monomer 1 is piperazine, monomer 2 is 2,6-Dichloro purine. The characteristic peaks at 1636 cm^{-1} in monomer 1 and 1585 cm^{-1} in COP-PD both represent the stretching vibration of C=N and the skeleton vibration of the benzene ring.

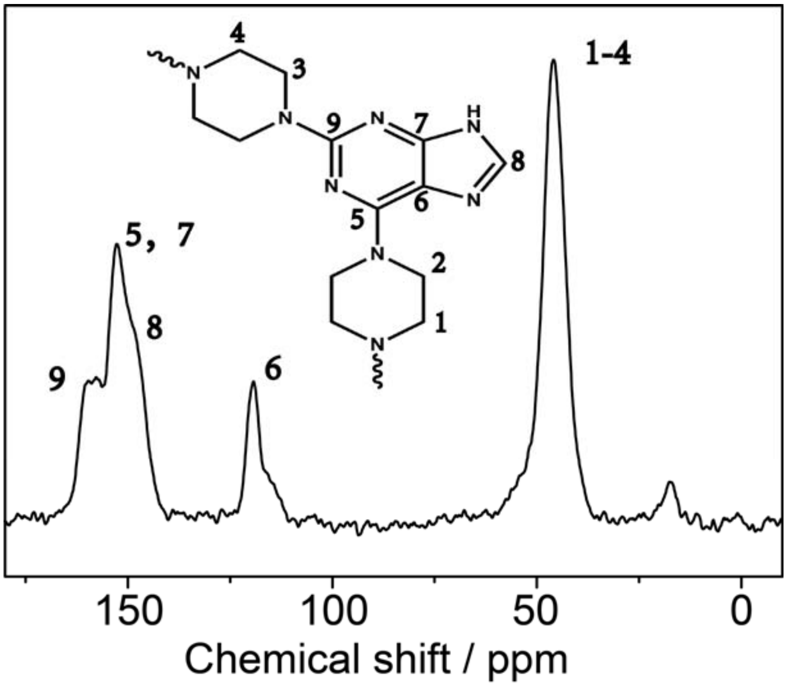

Figure S2. Solid-state ^{13}C CP MAS NMR spectra of COP-PD.

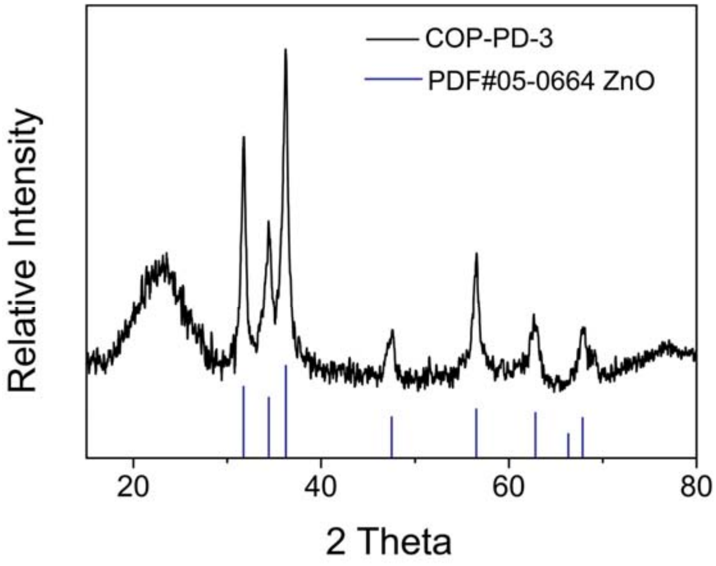

Figure S3. XRD patterns of COP-PD-3

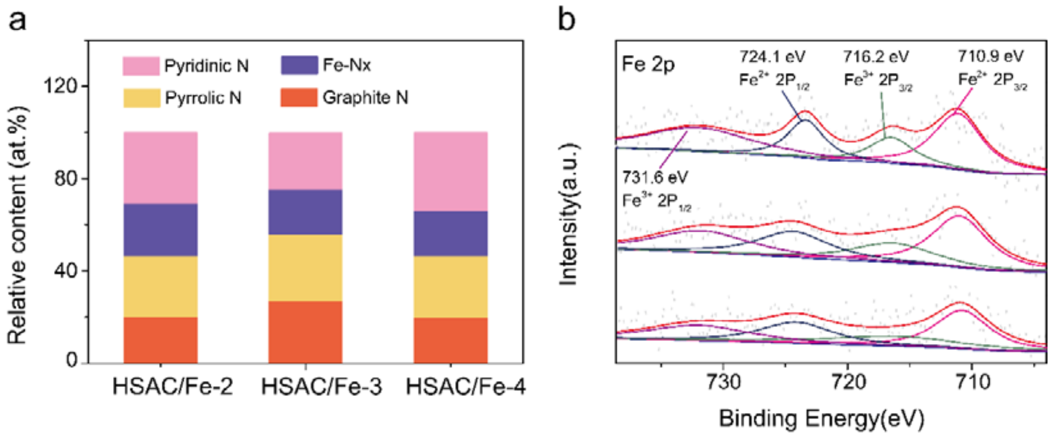

Figure S4. a) The relative contents of different types of N from high resolution XPS spectra of N1s. b) High resolution XPS spectra of Fe2p.

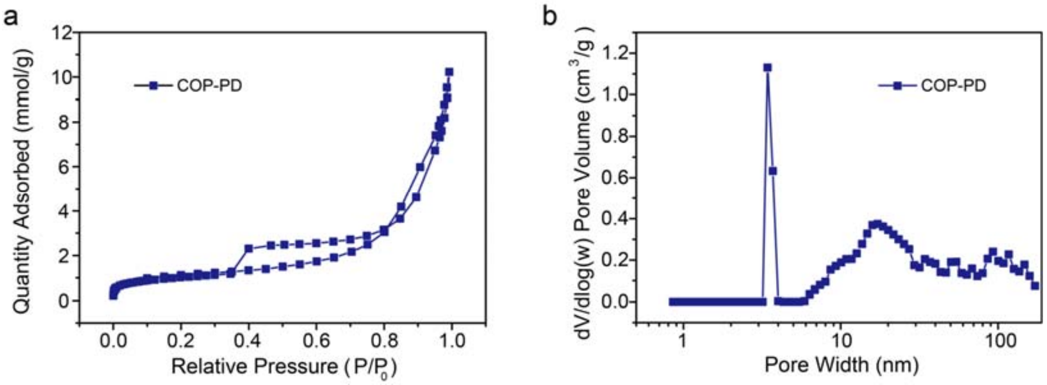

Figure S5. BET characterization of COP-Fe-Zn. a) nitrogen adsorption-desorption isotherms. b) The corresponding pore size distributions.

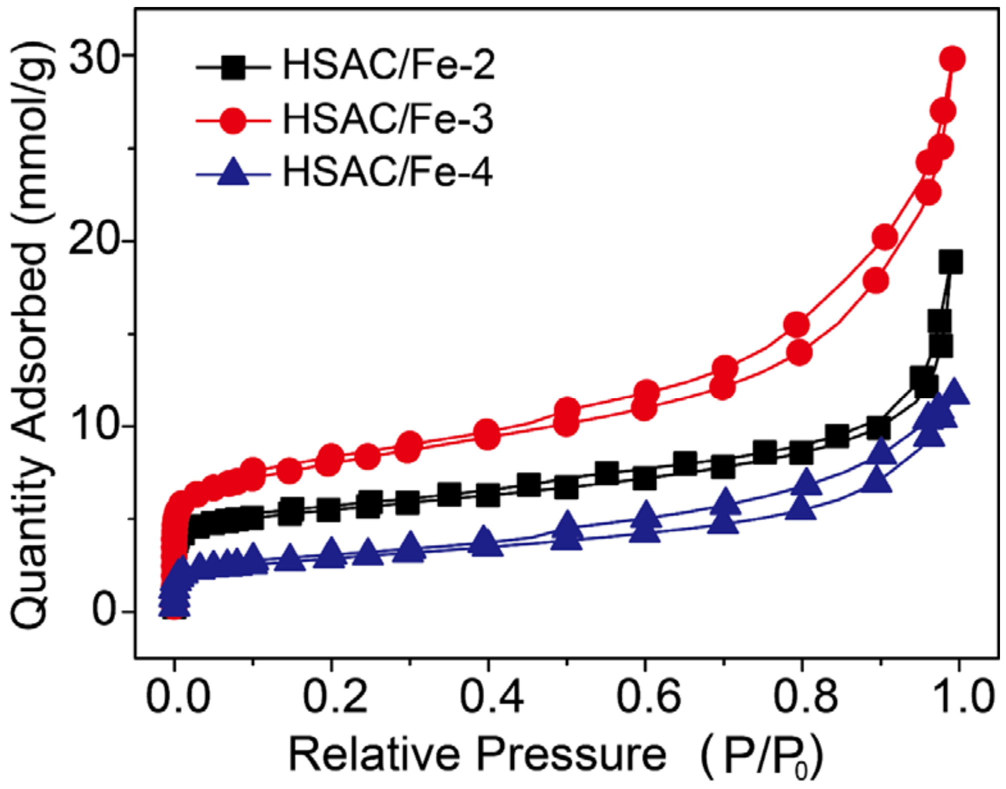

Figure S6. BET characterization, nitrogen adsorption-desorption isotherms.

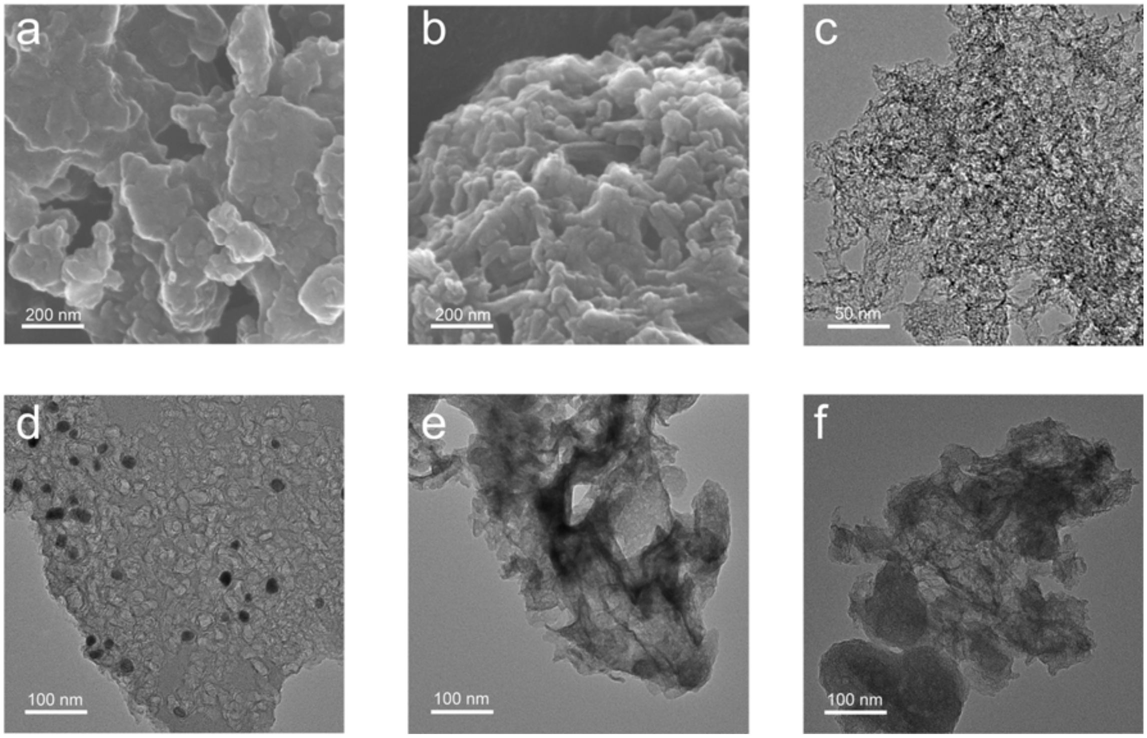

Figure S7. SEM image of a) COP-PD and b) HSAC/Fe-3.c) HRTEM image of HSAC/Fe-3. TEM image of d) HSAC/Fe-0, e) HSAC/Fe-2 and f) HSAC/Fe-4.

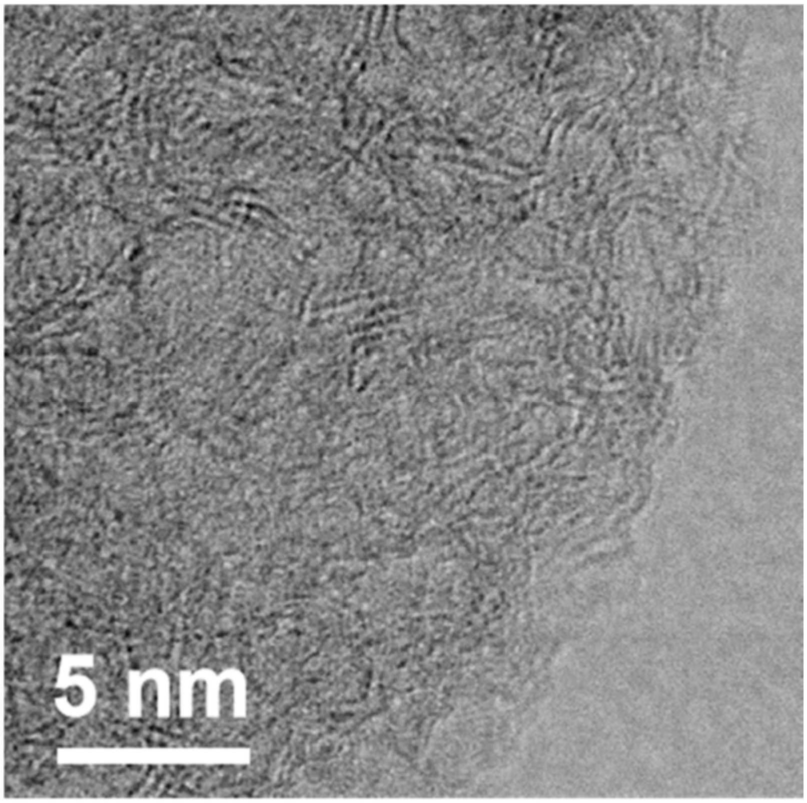

Figure S8. ABF-STEM image

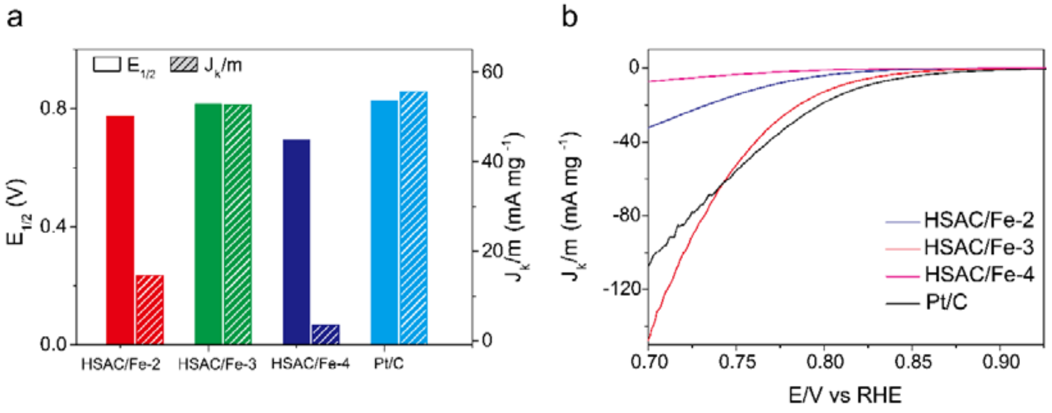

Figure S9. a) $E_{1/2}$ and mass activity at 0.75 V, b) mass activity curves of HSAC/Fe-2, HSAC/Fe-3, HSAC/Fe-4 and Pt/C.

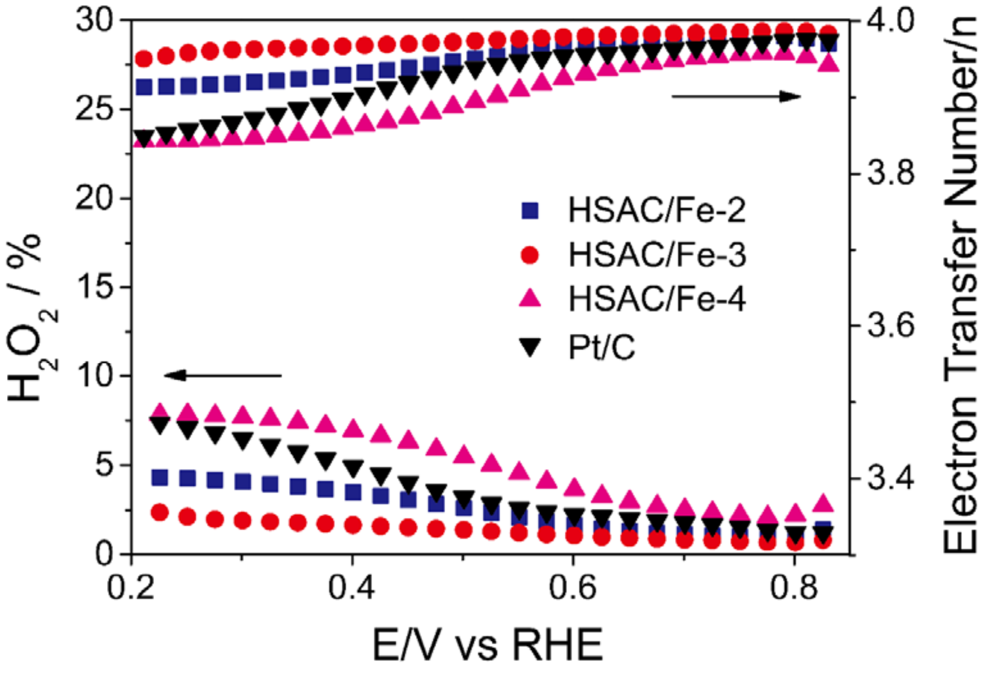

Figure S10. Peroxide yields and electron transfer numbers

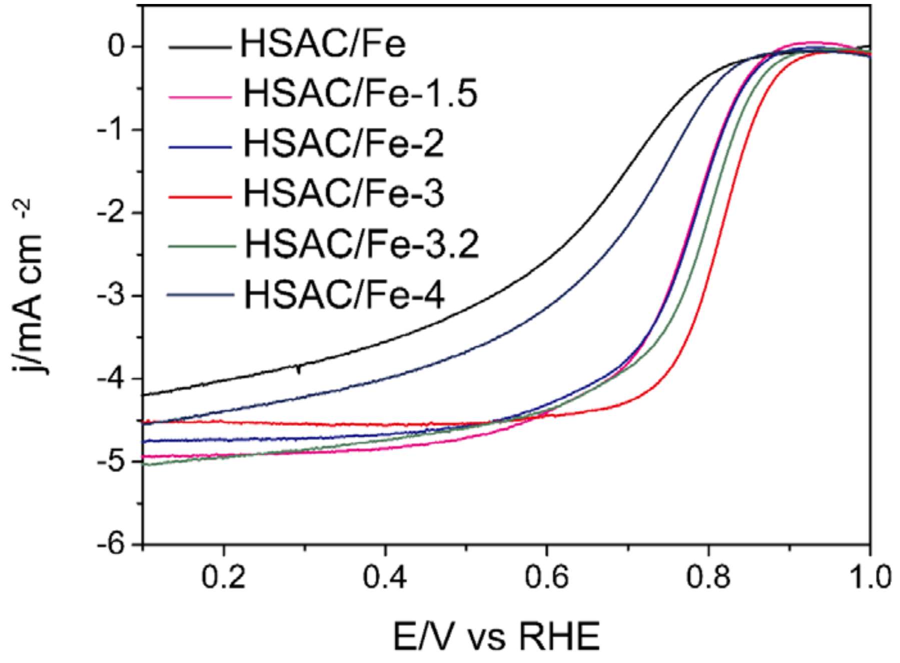

Figure S11. ORR polarization curves of catalysts with different Zn doping in O_2-saturated 0.5 M H_2SO_4 solution at a rotation rate of 1600 rpm.

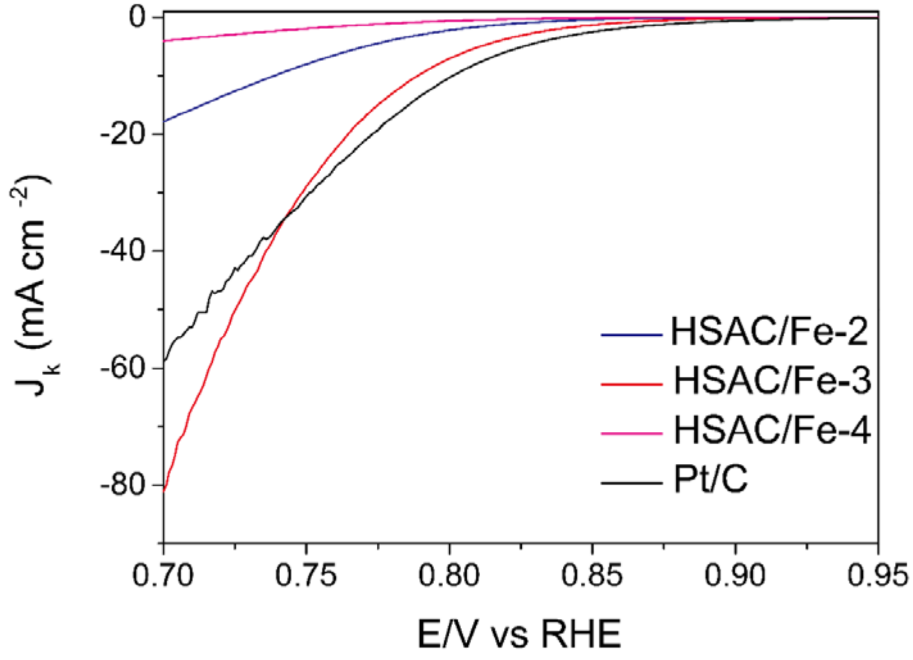

Figure S12. J_k for different catalyst

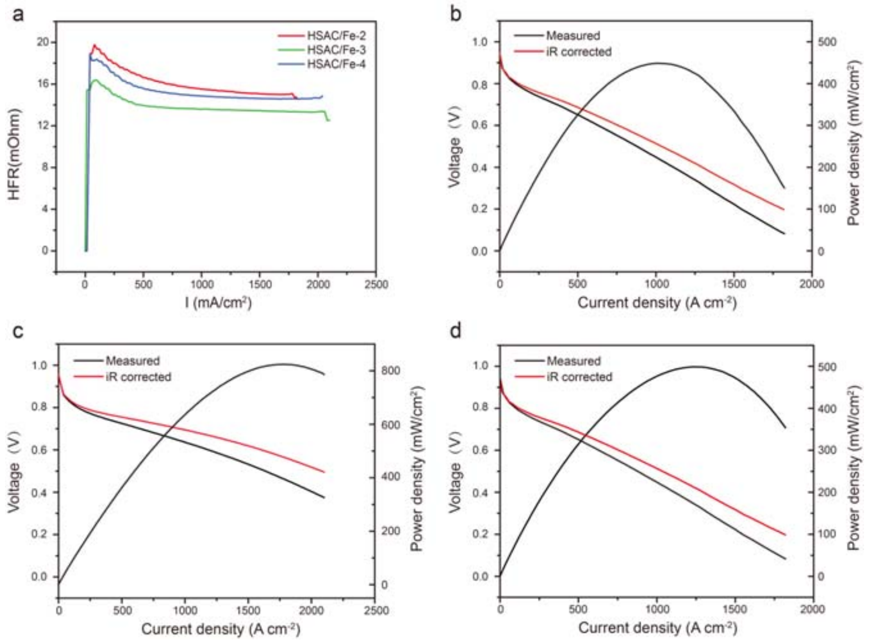

Figure S13. a) The HFR polarization for the three MEA. The iR-corrected polarization for the catalyst b) HSAC/Fe-2, c) HSAC/Fe-3 and d) HSAC/Fe-4, respectively.

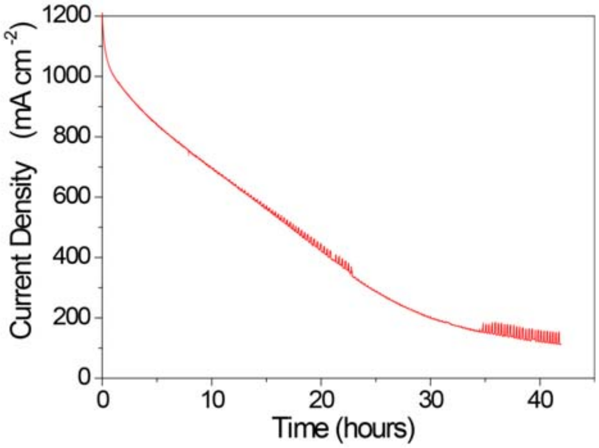

Figure S14. The stability of catalyst HSAC/Fe-3 in PEMFC.

Table S1. Surface elemental composition of catalysts determined by XPS.

Samples	C% (at/wt)	N% (at/wt)	O% (at/wt)	Fe% (at/wt)
**HSAC/Fe-2**	90.93/87.68	2.01/2.26	6.75/8.67	0.31/1.39
**HSAC/Fe-3**	89.09/85.45	1.93/2.16	8.69/11.10	0.29/1.29
**HSAC/Fe-4**	89.69/86.31	4.92/5.52	5.01/6.42	0.39/1.75

Table S2. Iron and zinc contents determined by ICP-OES

Samples	Fe wt%	Zn wt%
**HSAC/Fe-2**	3.29	1.96
**HSAC/Fe-3**	2.78	1.92
**HSAC/Fe-4**	3.37	2.58

Table S3. Specific surface area of different pore sizes in catalysts

Catalyst	S_{mic}/ $m^2\ g^{-1}$	S_{mes}/ $m^2\ g^{-1}$	S_{mac}/ $m^2\ g^{-1}$	$S_{mes}+S_{mac}$/ $m^2\ g^{-1}$	S_{mic}/S_{total}, %	S_{mes}/S_{total}, %	S_{total}/ $m^2\ g^{-1}$
HSAC/Fe-2	195.94	157.73	53.99	211.72	48.1	38.7	407.66
HSAC/Fe-3	209.57	297.67	99.49	397.16	34.5	49.1	606.73
HSAC/Fe-4	55.90	130.87	35.07	165.94	25.2	59.0	221.84

S_{mic}: specific surface area of micropore; S_{mes}: specific surface area of mesopores; S_{mac}: specific surface area of macropore;

Table S4. Fe K-edge EXAFS curves Fitting Parameters

Sample	Path	N	R(A)	σ^2(×10-3 A2)	ΔE_0(eV)	R-factor
HSAC/Fe-3	Fe-N(O)	5	2.01	1.33	5.5	0.012

N, corrdination number; R, distance between absorber and backscatter atoms; σ^2, the Debye-Waller factor value; ΔE_0 (eV), inner potential correction to account for the difference in the inner potential between the sample and the reference compound. R factor is used to value the goodness of the fitting.

Table S5. Comparison of ORR activities of various nonprecious catalysts in 0.5 M H_2SO_4 electrolyte.

Catalysts	Half-wave potential (V vs RHE)	Onset potential (V vs. RHE)	Rotation (rpm)	Ref.
Fe_2-Z_8-C	0.805	0.902	1600	[1]
1.5Fe-ZIF	0.88	/	900	[2]
20Mn-NC-second	0.8	/	900	[3]
Fe/SNC	0.77	<0.9	1600	[4]
Fe-PANI-EN-hydrogel	0.83	0.95	900	[5]
Co-NPs/HNCS	0.773	~0.88	1600	[6]
Fe-ZIF(50 nm)	0.85	~0.96	900	[7]
26AMP-ex	0.75	~0.88	1600	[8]
5% Fe-N/C	0.735	0.861	1600	[9]
Fe-N-CC	0.52	0.8	1600	[10]
HCS-A	~0.75	~0.85	900	[11]
Fe-N-C-Phen-PANI	0.8	~0.9	900	[12]
Fe-N/C NNs	0.662	0.858	1600	[13]
Co-N-C@F127	0.84	/	900	[14]
FeNC	0.75	~0.86	1600	[15]
Fe-N-C/VA-CNT	0.79	0.97	1600	[16]

Co-N-GA	0.73	0.88	900	[17]
Co-N-C@F127	0.8	0.93	900	[18]
**HSAC/Fe-3**	0.814	0.94	1600	This work

Table S6. Comparison of Power density of various catalysts in PEMFC.

**Catalysts**	**Power density**	**Ref**
CoNC-ArNH3	826 mW cm^{-2}	[19]
SA-Fe/NG	823 mW cm^{-2}	[20]
ZIF′-FA-CNT-p	820 mW cm^{-2}	[21]
Pt3Sc/PECNTs	760 mW cm^{-2}	[22]
Fe2(1.5%)–N–C2-900	540 mW cm^{-2}	[23]
C-FeHZ8@g-C3N4-950	628 mW cm^{-2}	[24]
Octahedral PtNi/C	881.6 mW cm^{-2}	[25]
Pt/TNTS-Mo	520 mW cm^{-2}	[26]
Pd@Pt3Co/C	854 mW cm^{-2}	[27]
**HSAC/Fe-3**	824 mW cm^{-2}	This work

References

[1] Q. Liu, X. Liu, L. Zheng and J. Shui, *Angew. Chem. Int. Ed.* **2018**, *57*, 1204.

[2] H. Zhang, H. T. Chung, D. A. Cullen, S. Wagner, U. I. Kramm, K. L. More, P. Zelenay and G. Wu, *Energ. Environ. Sci.* **2019**, *12*, 2548.

[3] J. Li, M. Chen, D. A. Cullen, S. Hwang, M. Wang, B. Li, K. Liu, S. Karakalos, M. Lucero, H. Zhang, C. Lei, H. Xu, G. E. Sterbinsky, Z. Feng, D. Su, K. L. More, G. Wang, Z. Wang and G. Wu, *Nat. Catal.* **2018**, *1*, 935.

[4] H. Shen, E. Gracia-Espino, J. Ma, K. Zang, J. Luo, L. Wang, S. Gao, X. Mamat, G. Hu, T. Wagberg and S. Guo, *Angew. Chem. Int. Ed.* **2017**, *56*, 13800.

[5] Z. Qiao, H. Zhang, S. Karakalos, S. Hwang, J. Xue, M. Chen, D. Su and G. Wu, *Appl. Catal. B-Environ.,* **2017**, *219*, 629.

[6] Y. Han, Y. G. Wang, W. Chen, R. Xu, L. Zheng, J. Zhang, J. Luo, R. A. Shen, Y. Zhu, W. C. Cheong, C. Chen, Q. Peng, D. Wang and Y. Li, *J. Am. Chem. Soc.* **2017**, *139*, 17269.

[7] H. Zhang, S. Hwang, M. Wang, Z. Feng, S. Karakalos, L. Luo, Z. Qiao, X. Xie, C. Wang, D. Su, Y. Shao and G. Wu, *J. Am. Chem. Soc.* **2017**, *139*, 14143.

[8] Y. Chen, R. Gokhale, A. Serov, K. Artyushkova and P. Atanassov, *Nano Energy* **2017**, *38*, 201.

[9] Q. Lai, L. Zheng, Y. Liang, J. He, J. Zhao and J. Chen, *ACS Catal.* **2017**, *7*, 1655.

[10] G. A. Ferrero, K. Preuss, A. Marinovic, A. B. Jorge, N. Mansor, D. J. Brett, A. B. Fuertes, M. Sevilla and M. M. Titirici, *ACS Nano* **2016**, *10*, 5922.

[11] P. Xu, J. Zhang, G. Jiang, F. Hassan, J.-Y. Choi, X. Fu, P. Zamani, L. Yang, D. Banham, S. Ye and Z. Chen, *Nano Energy* **2018**, *51*, 745.

[12] X. Fu, P. Zamani, J. Y. Choi, F. M. Hassan, G. Jiang, D. C. Higgins, Y. Zhang, M. A. Hoque and Z. Chen, *Adv Mater* **2017**, *29*, 1604456.

[13] N. Wu, Y. Wang, Y. Lei, B. Wang, C. Han, Y. Gou, Q. Shi and D. Fang, *Sci. Rep.* **2015**, *5*, 17396.

[14] Y. He, S. Hwang, D. A. Cullen, M. A. Uddin, L. Langhorst, B. Li, S. Karakalos, A. J. Kropf, E. C. Wegener, J. Sokolowski, M. Chen, D. Myers, D. Su, K. L. More, G. Wang, S. Litster and G. Wu, *Energ. Environ. Sci.* **2019**, *12*, 250.

[15] K. Mamtani, D. Jain, A. C. Co and U. S. Ozkan, *Energy & Fuels* **2017**, *31*, 6541.

[16] S. Yasuda, A. Furuya, Y. Uchibori, J. Kim and K. Murakoshi, *Adv. Funct. Mater.* **2016**, *26*, 738.

[17] X. Fu, J. Y. Choi, P. Zamani, G. Jiang, M. A. Hoque, F. M. Hassan and Z. Chen, *ACS Appl. Mater. Inter.* **2016**, *8*, 6488.

[18] X. X. Wang, D. A. Cullen, Y. T. Pan, S. Hwang, M. Wang, Z. Feng, J. Wang, M. H. Engelhard, H. Zhang, Y. He, Y. Shao, D. Su, K. L. More, J. S. Spendelow and G. Wu, *Adv. Mater.* **2018**, *30*.

[19] L. Chen, X. Liu, L. Zheng, Y. Li, X. Guo, X. Wan, Q. Liu, J. Shang, J. Shui, *Appl. Catal. B Environ*. **2019**, *256*, 117849.

[20] L. Yang, D. Cheng, H. Xu, X. Zeng, X. Wan, J. Shuic, Z. Xiang, D. Cao, *P. Natl. A. Sci.* **2018**, *26*, 6626.

[21] C. Zhang, Y. C. Wang, B. An, R. Huang, C. Wang, Z. Zhou, W. Lin, *Adv. Mater*. **2017**, *29*.

[22] M. S. Garapati, R. Sundara, *Int. J. Hydrogen Energ.* **2019**, *44*, 10951.

[23] L. Li, S. Shen, G. Wei, X. Li, K. Yang, Q. Feng, J. Zhang, *ACS Appl. Mater. Interfaces* **2019**, *11*, 14126.

[24] Y. Deng, B. Chi, X. Tian, Z. Cui, E. Liu, Q. Jia, W. Fan, G. Wang, D. Dang, M. Li, K. Zang, J. Luo, Y. Hu, S. Liao, X. Sun, S. Mukerjee, *J. Mater. Chem. A* **2019**, *7*, 5020.

[25] B. Li, J. Wang, X. Gao, C. Qin, D. Yang, H. Lv, Q. Xiao, C. Zhang, *Nano Res.* **2018**, *12*, 281.

[26] R. Alipour Moghadam Esfahani, L. M. Rivera Gavidia, G. García, E. Pastor, S. Specchia, *Renew. Energ.* **2018**, *120*, 209.

[27] K.C. Wang, H.C. Huang, C.H. Wang, *Int. J. Hydrogen Energ.* **2017**, *42*, 11771
